# Supplementary material for: Evaluation of sample pooling for screening of SARS CoV-2
Source: PLoS One. 2021 Feb 26;16(2):e0247767. doi: 10.1371/journal.pone.0247767 (PMC7909632; doi:10.1371/journal.pone.0247767)
Supplement: S1 Table — (DOCX) [file pone.0247767.s001.docx]

**S1 Table.** Ct values of the original positive sample (with low a Ct value highlighted in silver) and the pooling, this corresponds to figures 1A and 1B.

| Experiments | 1st experiment | | 2nd experiment | | 3rd experiment | | Average Ct values | |
| --- | --- | --- | --- | --- | --- | --- | --- | --- |
| Channels | FAM | VIC | FAM | VIC | FAM | VIC | FAM | VIC |
| Target genes | N gene | ORF1ab | N gene | ORF1ab | N gene | ORF1ab | N gene | ORF1ab |
| Original positive sample. AHRI-0105 | 28.59 | 30.97 | 28.64 | 30.90 | 28.58 | 30.44 | F 28.61 | 30.77 |
| Pooling (Postive:negative) |  |  |  |  |  |  |  |  |
| AHRI-0105 (1:1) | 29.62 | 31.66 | 29.28 | 31.42 | 29.92 | 31.74 | 29.61 | 31.61 |
| AHRI-0105 (1:2) | 31.22 | 33.11 | 30.37 | 32.50 | 30.23 | 32.93 | 30.61 | 32.85 |
| AHRI-0105 (1:3) | 33.88 | 34.05 | 35.67 | 35.30 | 34.26 | 35.45 | 34.60 | 34.94 |
| AHRI-0105 (1:4) | 32.36 | 33.68 | 31.26 | 33.44 | 30.82 | 33.40 | 31.48 | 33.51 |
| AHRI-0105 (1:5) | 31.80 | 34.12 | 31.87 | 33.90 | 31.81 | 33.74 | 31.83 | 33.92 |
| AHRI-0105 (1:6) | 31.29 | 33.30 | 31.09 | 33.55 | 31.24 | 33.18 | 31.21 | 33.34 |
| AHRI-0105 (1:7) | 31.43 | 34.11 | 31.84 | 34.03 | 31.86 | 33.39 | 31.71 | 33.85 |
| AHRI-0105 (1:8) | 32.70 | 35.19 | 32.11 | 34.33 | 31.96 | 34.18 | 32.26 | 34.57 |
| AHRI-0105 (1:9) | No Ct | 38.44 | No Ct | 38.88 | 32.97 | 35.26 | 32.97 | 37.53 |
