# Supplementary material for: Evaluation of sample pooling for screening of SARS CoV-2
Source: PLoS One. 2021 Feb 26;16(2):e0247767. doi: 10.1371/journal.pone.0247767 (PMC7909632; doi:10.1371/journal.pone.0247767)
Supplement: S2 Table — (DOCX) [file pone.0247767.s002.docx]

**S2 Table.** Ct values of the original positive sample (with a high Ct value silver highlighted) and the pooling

| Experiments | 1st experiment | | 2nd experiment | | 3rd experiment | | Average Ct values | |
| --- | --- | --- | --- | --- | --- | --- | --- | --- |
| Channels | FAM | VIC | FAM | VIC | FAM | VIC | FAM | VIC |
| Target genes | N gene | ORF1ab | N gene | ORF1ab | N gene | ORF1ab | N gene | ORF1ab |
| Original positive sample  AHRI-0073 | 33.70 | 36.70 | 34.95 | 37.64 | 34.47 | 37.46 | 34.83 | 37.27 |
| Pooling (postive:negative) |  |  |  |  |  |  |  |  |
| AHRI-0073 (1:1) | 35.29 | 37.90 | 35.58 | 38.01 | 35.38 | 37.43 | 35.75 | 37.78 |
| AHRI-0073 (1:2) | 35.83 | 38.32 | 36.00 | 38.24 | 36.02 | 37.43 | 36.32 | 38.00 |
| AHRI-0073 (1:3) | 35.90 | 38.74 | 35.09 | 38.35 | 37.09 | 37.70 | 37.04 | 38.26 |
| AHRI-0073 (1:4) | 38.61 | 39.23 | 37.13 | 38.60 | 37.48 | 39.01 | 37.48 | 38.95 |
| AHRI-0073 (1:5) | 38.67 | 40 | 37.81 | 40 | 36.25 | 39.29 | 38.11 | 39.76 |
| AHRI-0073 (1:6) | 38.23 | 39.06 | No Ct | 39.21 | 37.71 | 40 | 37.42 | 39.42 |
| AHRI-0073 (1:7) | 36.38 | 39.42 | 36.93 | No Ct | 37.20 | 38.59 | 37.94 | 39.00 |
| AHRI-0073 (1:8) | 38.10 | No Ct | 39.29 | No Ct | 38.73 | 39.89 | 38.60 | 39.89 |
| AHRI-0073 (1:9) | 38.19 | No Ct | 39.41 | No Ct | 38.86 | No Ct | 38.82 | No Ct |
