# Supplementary material for: Evaluation of sample pooling for screening of SARS CoV-2
Source: PLoS One. 2021 Feb 26;16(2):e0247767. doi: 10.1371/journal.pone.0247767 (PMC7909632; doi:10.1371/journal.pone.0247767)
Supplement: S3 Table — (DOCX) [file pone.0247767.s003.docx]

**S3 Table.** Ct values of the original RNA positive sample (with a low Ct value highlighted in silver) and the pooling, this corresponds to figures 2A and 2B

| Channels | FAM | VIC | Difference in Ct values (the original positive minus pool Ct value) | |
| --- | --- | --- | --- | --- |
| Target genes | N gene | ORF1ab | N gene | ORF1ab |
| Original RNA positive sample. AHRI-0105 | 27.74 | 29.18 |  |  |
| Pooling (postive:negative) |  |  |  |  |
| AHRI-0105 (1:1) | 29.27 | 30.41 | 1.53 | 1.23 |
| AHRI-0105 (1:2) | 29.47 | 30.89 | 1.73 | 1.71 |
| AHRI-0105 (1:3) | 29.75 | 31.29 | 2.01 | 2.11 |
| AHRI-0105 (1:4) | 29.82 | 31.83 | 2.08 | 2.65 |
| AHRI-0105 (1:5) | 30.19 | 32.35 | 2.45 | 3.17 |
| AHRI-0105 (1:6) | 30.07 | 32.3 | 2.33 | 3.12 |
| AHRI-0105 (1:7) | 30.31 | 32.32 | 2.57 | 3.14 |
| AHRI-0105 (1:8) | 30.41 | 32.51 | 2.67 | 3.33 |
| AHRI-0105 (1:9) | 30.93 | 32.63 | 3.19 | 3.45 |
