# Supplementary material for: Evaluation of sample pooling for screening of SARS CoV-2
Source: PLoS One. 2021 Feb 26;16(2):e0247767. doi: 10.1371/journal.pone.0247767 (PMC7909632; doi:10.1371/journal.pone.0247767)
Supplement: S4 Table — (DOCX) [file pone.0247767.s004.docx]

**S4 Table.** Ct values of the original RNA positive sample (with a medium Ct value highlighted in silver) and the pooling, this corresponds to figures 3A and 3B

| Channels | FAM | VIC | Difference in Ct values (the original positive minus pool Ct value) | |
| --- | --- | --- | --- | --- |
| Target genes | N gene | ORF1ab | N gene | ORF1ab |
| Original RNA positive sample. AHRI-0066 | 33.91 | 35.63 |  |  |
| Pooling (postive:negative) |  |  |  |  |
| AHRI-0066 (1:1) | 34.39 | 36.58 | 0.48 | 0.95 |
| AHRI-0066 (1:2) | 35.66 | 37.17 | 1.75 | 1.54 |
| AHRI-0066 (1:3) | 34.89 | 36.92 | 0.98 | 1.29 |
| AHRI-0066 (1:4) | 34.72 | 38.86 | 0.81 | 3.23 |
| AHRI-0066 (1:5) | 36.01 | 37.18 | 2.10 | 1.55 |
| AHRI-0066 (1:6) | 36.11 | 37.99 | 2.20 | 2.36 |
| AHRI-0066 (1:7) | 39.23 | 38.26 | 5.32 | 2.63 |
| AHRI-0066 (1:8) | 37.44 | 38.83 | 3.53 | 3.20 |
| AHRI-0066 (1:9) | 38.39 | 37.28 | 4.48 | 1.65 |
