# Supplementary material for: Evaluation of sample pooling for screening of SARS CoV-2
Source: PLoS One. 2021 Feb 26;16(2):e0247767. doi: 10.1371/journal.pone.0247767 (PMC7909632; doi:10.1371/journal.pone.0247767)
Supplement: S5 Table — (DOCX) [file pone.0247767.s005.docx]

| Disease prevalence rate (%) | Optimal sample pool size | Expected number of tests reduced (%) | Expected increase in testing efficiency (%) |
| --- | --- | --- | --- |
| 1 | 10 | 81 | 426 |
| 2 | 8 | 73 | 270 |
| 3 | 6 | 67 | 203 |
| 4 | 6 | 62 | 163 |
| 5 | 5 | 58 | 138 |
| 6 | 5 | 54 | 117 |
| 7 | 4 | 50 | 100 |
| 8 | 4 | 47 | 89 |
| 9 | 4 | 44 | 79 |
| 10 | 4 | 41 | 69 |
| 11 | 4 | 38 | 61 |
| 12 | 4 | 36 | 56 |
| 13 | 3 | 33 | 49 |

**S5 Table. A comparison of the influence of optimal sample pool size and disease prevalence rate on test efficiency**
